# Insights from transgenic mouse models of PyMT-induced breast cancer: recapitulating human breast cancer progression in vivo

**DOI:** 10.1038/s41388-020-01560-0

**Published:** 2020-11-24

**Authors:** Sherif Attalla, Tarek Taifour, Tung Bui, William Muller

**Affiliations:** 1 Department of Biochemistry, McGill University, Montreal, QC H3A 1A3 Canada; 2 Goodman Cancer Research Centre, McGill University, Montreal, QC H3A 1A3 Canada; 3 Faculty of Medicine, McGill University, Montreal, QC H3A 1A3 Canada

**Keywords:** Breast cancer, Cancer models, Molecular biology

## Abstract

Breast cancer is associated with the second highest cancer-associated deaths worldwide. Therefore, understanding the key events that determine breast cancer progression, modulation of the tumor-microenvironment and metastasis, which is the main cause of cancer-associated death, are of great importance. The mammary specific polyomavirus middle T antigen overexpression mouse model (MMTV-PyMT), first published in 1992, is the most commonly used genetically engineered mouse model (GEMM) for cancer research. Mammary lesions arising in MMTV-PyMT mice follow similar molecular and histological progression as human breast tumors, making it an invaluable tool for cancer researchers and instrumental in understanding tumor biology. In this review, we will highlight key studies that demonstrate the utility of PyMT derived GEMMs in understanding the molecular basis of breast cancer progression, metastasis and highlight its use as a pre-clinical tool for therapeutic discovery.

## Introduction

**Fig. 1:**
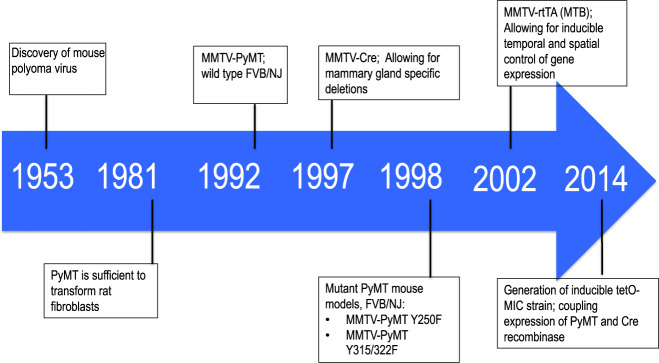
Notable advances in GEMMs of breast cancer. The unenveloped double stranded murine polyomavirus was discovered in 1953. The viral antigen with transformative capacity, the middle T (PyMT), was identified in 1981. Our lab published the first highly metastatic mammary tumor model expressing PyMT in the mammary gland in an FVB/NJ background. Introduction of MMTV-Cre to the field of oncomice in 1997 allowed for conditional ablation within the mammary epithelium. Mutant PyMT strains in 1998 were instrumental in dissection of PyMT associated signaling. The MTB strain was introduced in 2002 which is used to drive expression of the PyMT coupled to Cre recombinase in the tetO-MIC published in 2014.

Breast cancer is the most common cancer in women and is responsible for the second highest number of cancer-associated deaths [1]. The development of various Genetically Engineered Mouse Models (GEMMs) has been instrumental in elucidating the molecular events involved in breast cancer initiation, progression and metastasis (Fig. 1). The MMTV-PyMT (polyomavirus middle T antigen) GEMM (also known as MMTV-PyVmT or MMTV-MT), has revolutionized the field of oncomice and, to this day, remains the most commonly used GEMM in the field of cancer research due to the rapid development of multifocal tumors, extensive lung metastasis and its availability from commercial sources [2, 3]. This model has been used to study several aspects of breast cancer including; initiation, histological and molecular progression, metastasis and, more recently with the rise of immunotherapies, cancer immunology. Despite not being a human oncogene, PyMT mimics the signaling of receptor tyrosine kinases which are commonly activated in many human malignancies including breast cancer.

In this review, we will discuss the use of PyMT models to assess various angles of tumor progression, highlighting key studies and findings relating to the human disease. Finally, we will review several key therapeutic interventions and how these models were instrumental in their advancement to the clinic.

## The PyMT GEMMs faithfully recapitulate human breast cancer progression

**Fig. 2:**
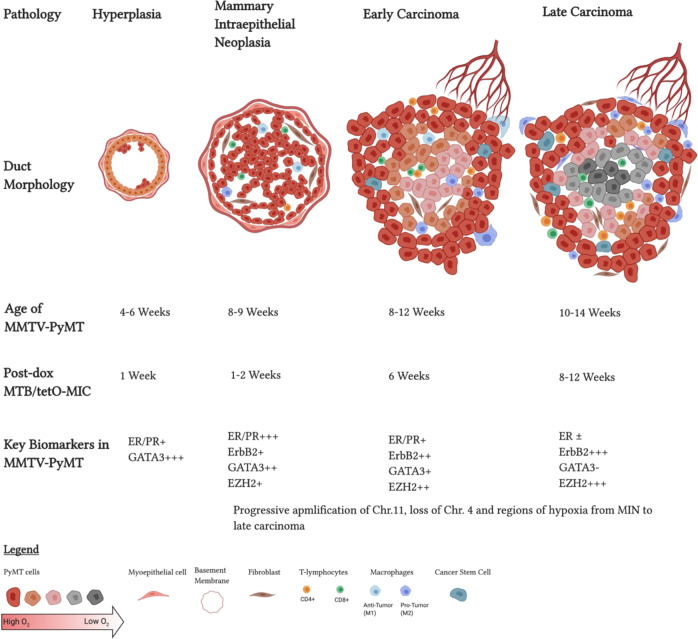
Progression of PyMT GEMMs. Both PyMT driven mouse models progress through the four main stages of cancer, akin to human tumors. Tumorigenesis starts with abnormal proliferation and hyperplasia, which then progresses through adenomas, mammary intraepithelial neoplasia (MIN), early carcinomas and culminates in late carcinomas that metastasizes to the lungs, forming adenocarcinomas in the lung parenchyma. Time points at which 50% of the animals have these pathologies for either MMTV-PyMT or MTB/tetO-MIC in weeks. Biomarkers associated with breast cancer shown at corresponding time points based off the MMTV-PyMT. Created with Biorender.com.

First described by our group in 1992, the MMTV-PyMT FVB/NJ strain made use of the murine mammary tumor virus long terminal repeat promoter (MMTV-LTR) to drive expression of **P**ol**y**omavirus **M**iddle **T** antigen (PyMT) [2, 4, 5]. Expressing the middle T oncogene in the mammary epithelia resulted in rapid transformation and generation of multifocal tumors that readily metastasize to the lungs [2]. Tumors arising from the luminal cells of the mammary gland progress through distinct histological stages that mimic human ductal breast cancer progression [3]. Nulliparous mice from founder #634 develop hyperplasia at 4 weeks of age, which progress through adenomas, mammary intraepithelial neoplasia (MIN), early and late carcinomas before metastasizing to the lungs, forming adenocarcinomas in the lung parenchyma (Fig. 2) [2, 3]. Gene expression profiling revealed that PyMT tumors cluster with the luminal B subtype of human breast cancers [6]. They also display loss of estrogen receptor (ER) and progesterone receptor expression (PR), overexpression of ErbB2 and cyclin D1 as the disease progresses (Fig. 2), mimicking human breast cancers with poor prognosis [3, 7]. Loss of ER is common in breast tumors resistant to endocrine therapy, as well as in recurrent cancers [8]. End-stage tumors also display robust infiltration by various immune cells such as macrophages and T cells which contribute to progression and metastasis [3, 9–11]. Together these pathological and molecular analyses make this GEMM a clinically relevant tool that models the human disease.

Several subsequent studies revealed that the expression of the PyMT oncogene is coupled to additional biochemical and genetic events that drive tumorigenesis and metastasis [12]. To that end, genomic analysis and whole exome sequencing was performed on the arising tumors [13, 14]. While these tumors maintain their diploid status, amplifications and deletions have been reported [13]. For example, amplifications of chromosome 11 are common in these tumors [13–17]. Chromosome 11 is orthologous to chromosome 17 in humans, which is commonly amplified and associated with increased PI3K activity due to amplification of the ErbB2 locus [15–18]. Expression of chromosome 11 genes such as *ErbB2*, *Septin9*, *Col1a1*, and *Chad* are elevated in PyMT tumors, as is the case in human breast cancers [3, 13, 19]. Although less common, deletions have been detected in chromosome 4, which codes for several tumor suppressors [13, 15]. Single nucleotide variants and activating mutations were detected through whole-exome studies and were found to be very common in naïve PyMT tumors and drive tumor progression and metastasis [14]. Moreover, whole genome sequencing detected several mutations in PyMT tumors [19]. For example, identical mutations in the gene coding for the receptor tyrosine phosphatase *Ptprh* are seen in 81% of PyMT tumors [19]. This mutation renders the phosphatase incapable of dephosphorylating EGFR, causing its constitutive activation [19, 20]. Similar *Ptprh* mutations were detected in non-small cell lung cancers and showed sensitivity to tyrosine kinase inhibitors [20]. Furthermore, our group has recently identified functionally active point mutations within the *Mtor* gene in RHEB GTPase deficient PyMT tumors [21]. Significantly, many of these *Mtor* mutations have also been found in human tumors [21]. It is important to note that the amplifications, deletions and genetic changes start during the hyperplasia stage and continue to change as the tumors advance to later stages, indicating progressive molecular changes that parallel the histological changes in this model (Fig. 2) [22]. Overall, these findings support the concept that full malignant transformation requires additional genetic events in addition to PyMT’s expression which leads to generation of transcriptionally heterogeneous tumors [19]. The fact these aberrations are also detected in human breast cancer reinforces the view that tumor progression in this GEMM faithfully recapitulates the many complex stages and heterogeneity of human breast cancer within a very short latency.

## The PyMT is a potent oncogene that associates with several cancer specific signaling pathways

The original rationale for studying the transforming potential of PyMT in the mammary epithelium derived from its capacity to activate a number of key cell-intrinsic signaling pathways involved in human breast cancer progression. While the PyMT oncogene lacks a kinase domain, it mimics an activated receptor tyrosine kinase through its association and interaction with a number of signal transducers such as SRC kinases, ShcA, phosphatidylinositol 3-kinase (PI3K) and phospholipase C-gamma 1 (PLCγ 1) among others [23–26]. These interactions depend on PyMT’s ability to form a complex with the scaffolding protein PP2A, which then recruits SRC family kinases [27]. PyMT can be phosphorylated by SRC kinase on tyrosine 315, 322, and 250 [28]. Each phosphorylation event activates a different pathway, which contributes to transformation. Phosphorylation at residue 250 recruits the SchA adapter protein that can recruit and activate the Ras/MAPK pathway [24, 29]. Tyrosine 315 is known to activate the PI3K pathway while phosphorylation of the 322 residue recruits PLCγ 1 [25, 30]. SRC recruitment is essential for PyMT mediated signaling and transformation as full-body knockout of *c-Src* rendered PyMT incapable of generating mammary tumors [31]. Subsequent analyses of the full-body knockout *c-Src* strain revealed major dysfunction in ERα signaling, indicating interplay between epithelial specific and stromal interactions [32]. More recently, in vivo and in vitro epithelial ablation of *c-Src* revealed that SRC is associated with cell cycle progression [33].

The importance of the PI3K and SchA signaling pathways was highlighted by the generation of mutant MMTV-PyMT (Y250F, Y315/322F) strains [34]. Mice expressing Y250F PyMT are incapable of binding SchA while Y315 and Y322 mutant impairs PI3K signaling [34]. GEMMs expressing the mutant PyMT exhibited a delay in tumor onset and impaired metastatic progression [32]. Remarkably, a proportion of the metastatic mammary tumors that arose in PyMT Y250F GEMM possessed either point mutations or in frame deletions that restored the NPTY motif required for ShcA binding, indicating a strong selective pressure for retention of this signaling pathway [34]. Consistent with this, crossing MMTV-PyMT and various knock-in models of ShcA mutants resulted in profound impact on tumor induction [35]. Detailed molecular analyses revealed that different ShcA mutants correlated with differential activation of the downstream STAT1 and STAT3 transcription factors [36]. In contrast, the PyMT Y315/322F GEMM did not revert back to wild-type form, but rather upregulated the expression of ErbB2 and ErbB3 which in turn increased PI3K signaling indicating that restoration of this key signaling pathways can also occur in an indirect fashion [34].

## Second generation inducible strain offers temporal and spatial regulation of PyMT expression, ablation of conditional alleles and an in vivo model of tumor induction

The MMTV-PyMT model has been utilized together with either full-body knockouts or with mammary specific expression of Cre recombinase (MMTV-Cre) [37] along with conditional alleles to knockout various proto-oncogenes and examine their effects on mammary tumorigenesis and metastasis. While full-body knockout studies were very informative and allowed researchers to elucidate the roles of different pathways during tumorigenesis, as well as identify novel targets for therapeutic intervention, these studies were very limited and had several drawbacks. As highlighted by c-*Src* studies, germline ablation impacts multiple tissues and may even be lethal in some cases [31, 32]. Many genes involved in cancer progression are also important for normal mammary gland development, thus presenting a confounding variable for breast cancer studies [32].

**Fig. 3:**
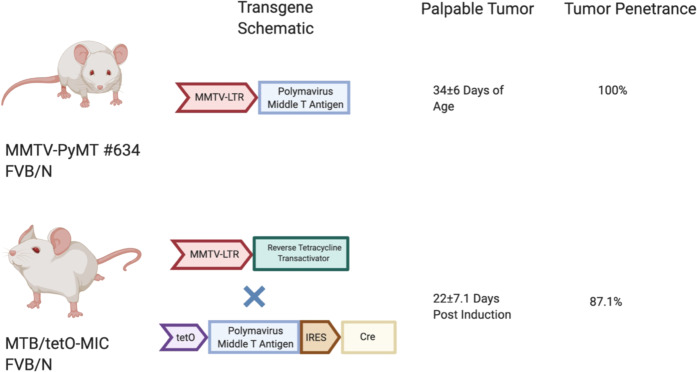
Comparison of PyMT GEMMs. Both strains were initially generated on an FVB/N background. Line #634 is the commonly used line for the MMTV-PyMT, which develop palpable tumors by 34 ± 6 days of age at a 100% penetrance. The MTB/tetO-MIC requires a genetic cross between the MTB (MMTV-rtTA) and the tetO-MIC strain. Bigenic mice develop multifocal palpable tumors at 22 ± 7.1 days post doxycycline induction in drinking water. Only 87.1% of bigneics are expected to develop tumors. Created with Biorender.com.

Conditional knockouts using MMTV-Cre is a common way to bypass embryonic lethality and multiple tissue effects. However, selective pressure in MMTV-PyMT/MMTV-Cre bigenics, driven by stochastic expression of the MMTV promoter, allows for generation of escapee tumors [38]. These tumors would express the PyMT oncogene and lack Cre recombinase expression and would therefore retain expression of the conditional allele. The generation of such escapee tumors renders interpretation of the role of critical signaling molecule in tumor induction difficult to assess. To address this issue, a doxycycline inducible model of PyMT linked to Cre recombinase, referred to as the tetO-MIC (tetO-Py**M**T-**I**RES-**C**re), was generated [39]. Using an MMTV driven reverse Tetracycline Transactivator strain (MTB) [40], doxycycline-dependent expression of PyMT and Cre recombinase in the mammary epithelial cell can be achieved in MTB/tetO-MIC bigenics (Fig. 3). Upon induction, these mice progress through the same histopathological stages seen in MMTV-PyMT and are highly metastatic to the lung [39]. Thus, this model offers the same advantages and human relevance of MMTV-PyMT mice with the ability to coincidentally ablate additional genes with no escapee phenomenon [21, 41, 42]. Another interesting facet of the inducible MTB/tetO-MIC strain is that PyMT is expressed only in the adult animal and therefore, is not recognized as self-antigen but a tumor specific antigen that is recognized and targeted by the immune system. In this regard, it is interesting to note that, in contrast to the original MMTV-PyMT strain where mammary tumor penetrance is 100% [2], although all female MTB/tetO-MIC develop mammary epithelial hyperplasias only 87% of these animals develop mammary tumors due to immune-selective pressure [39, 41, 42].

Further confirmation of the MTB/tetO-MIC model undergoing active immune surveillance stems from crosses with the conditional *Stat3* strain. While epithelial ablation of STAT3 did not impact the initial hyperplastic expansion of the epithelial tree, the nascent lesions were eliminated by massive immune response involving both anti-tumor macrophage and T cell populations [41]. Moreover, only 20% of mammary gland deficient STAT3 animals developed focal mammary tumors at a significantly delayed onset compared to their wildtype counterparts [41]. Interestingly, the STAT3 tumors that escaped immune surveillance failed to metastasize to distal organs, indicating that STAT3 was critical for the metastatic phase of tumor induction [41]. This key study represents the first GEMM where the various phases of immune editing, including tumor elimination, immune equilibrium, and immune escape can be mechanistically dissected [41, 43]. It is important to mention that STAT3 is an essential transcription factor that affects a wide variety of cell-intrinsic and extrinsic signaling pathways that together contribute to tumor cell proliferation and metastasis [44]. PyMT cells lacking STAT3 are capable of growing in immune-compromised mice [36]. However, only around 25% of mice injected with STAT3 deficient PyMT cells develop tumors in an immune-competent background, highlighting STAT3’s essential role in PyMT mediated immune suppression [36].

Using primary mammary epithelium from the MTB/tetO-MIC model, 3D organotypic cultures were developed that could be induced to express the PyMT oncogene [45]. Prior to doxycycline administration, luminal cells from the glands were cultured and formed a 3D structure with a lumen, recapitulating the architecture of the normal mammary gland [45]. Upon induction of PyMT expression, cells progressively filled the lumen [45]. This progressive filling of the duct is akin to tumor induction and progression from early neoplastic transformation to a ductal carcinoma in situ in human breast cancers [46]. This provides a tool to dissect the early players in mammary gland transformation using an in vitro model system. This tool can be used as a screening platform for inhibitors, as well as in combination with CRISPR/Cas9 medicated genetic ablation of genes of interest. Furthermore, MTB/tetO-MIC organoids can be adapted into co-culture systems with fibroblasts or individual components of the immune system such as macrophages in order to study the contribution of these cell types to tumor induction and progression.

Another aspect of the MTB/tetO-MIC strain is that it can be crossed into conditional, mutant knock-in strains. These knock-in mice could carry a mutant exon within intronic sequences and a loxP site flanking the wildtype exon [47]. Cre expression allows deletion of the wildtype exon and in-frame expression of the mutant version [47]. These models would be a valuable tool to assess the role of commonly seen mutations and their direct contribution to tumorigenesis in a controlled fashion.

## PyMT GEMMs as a platform to study molecular signaling driving tumor initiation and progression

The PyMT mouse model has also been extensively used to study the events that promote tumor initiation and mutations that predispose patients to breast cancer. A comprehensive review of the effects of different genes on tumor onset and metastasis is provided in Table 1 along with a brief description of its relevance and impact on the field. Cancer initiation describes the early genetic changes that drive a healthy cell into malignancy. It arises due genetic and epigenetic (discussed later) changes, mutations, amplifications or deletions that inactivate tumor suppressors or activate oncogenes.

**Table 1:** Genes affecting onset and progression in PyMT.

Gene	General function	Strain background	Effect on tumorigenesis	Relevance	Reference
HIF1α	Transcription factor, important for the ability of mammalian cells to respond to decreased oxygen levels.	FVB/NJ	Delayed tumor onset and decreased lung metastasis.	Presented evidence that Hif1α is important for cancer stem cells, and provided rationale for targeting it to prevent cancer recurrence.	[54, 55]
CCR6	Chemokine receptor for the ligand CCL20. Found on dendritic cells, T cells, B cells and NK cells.	C57BL/6J	Delayed tumor onset, due to defective initiation	Macrophages (especially M2) promote tumorigenesis and tumor initiation.	[61]
MMP8	Collagen degrading enzyme. Functions to modify the ECM	FVB/NJ	Accelerated tumor onset	Evidence that MMPs may have an anti-cancer role. MMP8 functions as a tumor suppressor. This is why their inhibitors may not be good in clinic	[50]
RAG1	Recombination activating gene 1. Essential for VDJ recombination. Knockout impairs B and T cell development. Mice lack an adaptive immune system	C57BL/6J	Accelerated tumor onset	Evidence that the adaptive immune system suppresses tumor formation and plays a role in immune surveillance.	[43]
IL15	Cytokine important for NK cell differentiation, proliferation and activation of T cells.	C57BL/6J	Accelerated onset and tumor growth	IL15 plays an important role in promoting an anti-tumor immune response. Early on in tumor formation, it stimulates 2 types of innate T lymphocytes, as well as CD8 and NK cells. Evidence of immune surveillance	[60]
STAT3	Major transcription factor. Regulates expression of genes important for survival, proliferation, differentiation and immune supression	FVB MTB/tetO-MIC	Delayed tumor onset but no effect on initiation. Tumor bearing mice were completely void of metastasis.	STAT3 is dispensable for tumor initiation but is essential for progression and metastasis. Also highlighted role of STAT3 in immune-suppression. The model recapitulates immunoediting	[41]
TBRII	Type II TGFβ receptor	FVB/NJ	Accelerated tumor onset	Absence of TGFβ signaling accelerates tumor formation and showed that TGFβ is a tumor suppressor	[105]
CSF-1	Cytokine that promotes macrophage differentiation and recruitment.	FVB/C57Bl/6J mixed	No change in tumor onset. Depletion of Tams led to decrease in pulmonary metastasis.	No change in initiation. Highlighted the role of macrophages in promoting breast cancer metastasis and progression.	[11]
MALAT1	Long non-coding RNA that is upregulated in tumors with high chance to metastasize	C57Bl/6J	No difference in tumor onset but slowed down tumor growth and decreased metastasis.	First in vivo evidence to show that this long non-coding RNA Is not essential for tumor initiation but regulates progression and metastasis.	[106]
ERBB3	Heterodimerizes with ErbB2 to induce proliferation, growth and survival	FVB/NJ	Delayed tumor onset and decreased metastasis	Activation of ErbB3 is required for transformation and thus it is a viable therapeutic target.	[66]
CTSB	Cathepsin B is a lysosomal cysteine protease that degrades ECM	FVB/NJ	Delayed tumor onset and decreased lung metastasis	CTSB is important for overcoming tumor dormancy and promoting tumor growth by degrading the ECM	[107]
EZH2	Methyltransferase, part of PRC2. Important for gene silencing.	FVB MTB-tetO-MIC	Delayed tumor onset and decreased metastasis	Highlighted context and subtype dependent role of Ezh2 in breast cancer progression	[42]
GCLM	Modifier subunit of glutamate cysteine ligase (GCL), which synthesizes glutathione.	C57BL/6J	Delayed tumor onset	Shows that synthesis of the antioxidant GSH facilitates tumor initiation but is not essential for later stages of cancer progression	[108]
PTHRP	Parathyroid hormone related protein, a secreted factor found to be elevated in breast cancers. It has a wide range of developmental functions and stimulates growth.	FVB/NJ	Delayed tumor onset, progression and decreased metastasis	First study to elucidate the tumor-promoting role of PTHRP in breast cancers, and highlighted it as a potential prognostic indicator or therapeutic target.	[109]
SNAIL1	Zinc finger Transcription factor, known to regulate EMT	FVB/NJ/C57Bl/6J mixed	Delayed tumor onset due to decreased proliferative capacity of TIC	Highlighted Snail1 as a major regulator of TIC proliferation and a potential target for therapies to inhibit cancer recurrence.	[59]
RHEB1	GTPase essential for activation of mTORC1 signaling	FVB/NJ MTB/tetO-MIC	Delayed onset due to reduced activity of mTORC1	Highlighted that mTORC1 signaling is essential for PyMT tumorigenesis. Arising mutations highlight the use of PyMT models in recapitulating genetic modulation as human tumors.	[21]

Deletions in the PyMT mice allowed identification of novel tumor suppressors, whose deletion accelerates tumor onset and their inhibition would be inadvisable in the clinic. One such tumor suppressor was the extracellular matrix-modifying enzyme MMP8. Matrix metalloproteinases (MMPs) have long been linked to metastasis and tumor progression due to their role in ECM degradation and cancer cell invasion [48]. MMPs were previously regarded as the key to cancer cells’ ability to spread to distant organs and several inhibitors were developed and tested in clinical trials [49]. Ablation of MMP8 in the MMTV-PyMT model accelerated tumor onset and increased incidence of lung metastasis [50]. This study was the first to show an in vivo evidence of an MMP acting as a tumor-suppressor and that its therapeutic inhibition would not be advisable [50]. Human studies have also shown that higher levels of MMP8 are correlated with good prognosis and better outcomes in breast cancers [50].

As tumors progress, they become deprived of oxygen, especially at the center of the tumor (Fig. 2) [51]. This phenomenon is known as hypoxia and induces the expression of hypoxia inducible factors (*Hif*s) [52]. HIF1α, in particular, is overexpressed in several types of cancers and is known to be associated with poor prognosis and decreased survival in cancer patients [53]. The levels of HIF1α progressively increase with tumor progression in the MMTV-PyMT model, mimicking human patients and making it an ideal model to study the roles of hypoxia on breast cancer progression, metastasis and therapy [54]. Mammary epithelial cell deletion of HIF1α in MMTV-PyMT mice significantly delayed tumor onset, slowed down tumor growth and caused a significant reduction in lung metastases [54, 55]. This study highlighted the role of this oxygen-dependent transcription factor in tumor initiation and progression to advanced disease. Deletion of HIF1α in vitro decreased the tumorigenic potential of PyMT cells, as well as their ability to form tumor-initiating cells (TICs) [54]. TICs and cancer stem cells are major concerns for breast cancer patients and are a major cause for cancer recurrence [56]. Cancer stem cells have been characterized in the PyMT models of breast cancers, commonly expressing markers such as CD24+CD29+CD49f+Sca-1^lo^ [57]. Moreover, the transcription factor GATA3 was identified as a tumor suppressor that inhibits proliferation of cancer stem cells [58]. Knockout of *Gata3* accelerates tumor onset due to the increased TIC capacity of luminal progenitor cells [58]. On the other hand, deletion of the zinc-finger transcription factor *Snail1* delayed tumor onset and caused a significant reduction in the proliferative capacity of TICs [59]. Thus, these studies highlight a dynamic balance between transcription factors that regulate TIC and show that PyMT models are ideal for the study of cancer stem cells and develop interventions against them that could prevent relapse and cancer recurrence.

In addition to TICs and cancer stem cells, the MTB/tetO-MIC strain, in particular, is a valuable tool to study tumor recurrence. Tumor-bearing mice stripped of doxycycline drinking water and provided with normal drinking water, show strong robust tumor regression to a virgin-like state [39]. However, these animals do eventually display doxycycline-independent tumor recurrence [39]. Recurrent tumors displayed various pathologies not seen in the pre-regression tumors and infrequent mutations in TP53 [39]. Thus, the MTB/tetO-MIC strain may be a valuable tool to study recurrent tumors that do not respond to the primary therapy since most recurrent tumors are no longer dependent on PyMT expression.

PyMT mouse models have also been used to study the roles of the immune system in cancer initiation and progression (see later). A germline knockout of RAG1, a key enzyme in VDJ recombination, eliminates the adaptive immune system (B and T cells) and accelerates tumor onset in the MMTV-PyMT (C57BL/6J) mouse [43]. Knocking out the interleukin IL15 also caused a similar acceleration in tumor onset [60]. Analysis of early lesions revealed that IL15 stimulates two separate populations of T cells, as well as natural killer and dendritic cells to target cancerous growth, thus highlighting it as a key tumor suppressive cytokine [60]. On the other hand, macrophages play a dual role in cancer initiation. While depletion of total macrophages through the knockout of Colony Stimulating Factor 1 (CSF1) had no effect on tumor onset, the depletion of alternatively activated macrophages (M2 polarized) through knockout of CCR6 delayed the onset of tumors [11, 61]. As discussed in the previous section, deleting STAT3 in the MTB/tetO-MIC model results in immune-system remodeling and complete elimination of hyperplastic growth, dramatically delaying tumor onset [41]. Collectively, these studies highlight the diverse and dynamic roles that cells of the innate and the adaptive immune system play in cancer initiation and progression. Further analysis is required to fully understand the complex interactions between cancers and different populations of immune cells, which will allow for development of more effective immunotherapies.

About 70% of breast cancers are ER positive, even at the metastatic setting [62]. The MMTV-PyMT has been used to assess the functionality of the ER. PyMT tumors are sensitive to the estrogen receptor modulator, tamoxifen, despite the low expression of nuclear ER in end stage tumors [63]. PyMT animals lacking the ER co-transcription factor Activated In Breast cancer 1 (AIB1) display delayed tumor onset and a reduction in pulmonary metastasis [64]. This suggests that the metastatic cascade of PyMT is driven, at least in part, by the ER [64]. Moreover, exogenous supplementation of estradiol to mice harboring PyMT tumors increases rate of tumor growth which was associated with increased vasculature organization [65]. On the other hand, similar to ~30% of breast cancers, advanced PyMT tumors express high levels of ErbB2 [3] Indeed, PyMT tumors respond to the EGFR/ErbB2 tyrosine kinase inhibitor lapatinib [66]. Furthermore, knockout of the ErbB3, the preferred heterodimerization partner of ErbB2, delayed tumor onset and impaired metastasis of MMTV-PyMT [66].

Ultimately, the cancer cell senses its extracellular matrix environment through multiple receptors, including integrin receptors, which in turn activates oncogenic downstream signaling pathways within the cancer cell [67]. The critical importance of integrin receptors in mammary tumor progression was highlighted through mammary epithelial ablation of the β1 integrin (CD29) in MMTV-PyMT [38]. These studies demonstrated that loss of β1 integrin in an emerging PyMT tumor resulted in a dramatic impairment of mammary tumorigenesis [38]. Significantly, the PyMT tumors that emerged retained expression of β1 integrin due to escapee from Cre mediated recombination (see earlier) highlighting the importance of β1 integrin in tumorigenesis. In addition to impacting the initiation phase of tumor progression, ablation of β1 integrin function in PyMT cell lines resulted in cell cycle arrest, inducing a state of cellular dormancy [38]. Consistent with the importance of integrin signaling in mammary tumor progression, mammary epithelial deletion of the integrin associated signaling protein, Focal Adhesion Kinase (FAK) resulted in a similar impact on PyMT tumor progression and induction of cellular dormancy [68]. Again, like β1integrin ablation studies, PyMT tumors that arose retained FAK expression due to escapee from Cre mediated recombination [68]. Future studies using the MTB/tetO-MIC model with these key regulators of tumor dormancy will provide critical insight into the molecular and cellular mechanisms governing emergence from tumor dormancy.

## PyMT tumor progression is associated with epigenetic modulations

In addition to mutations, amplifications and other genetic changes, epigenetic alterations have also been shown to be common in cancers, maybe even driving tumor development through epigenetic silencing of tumor suppressor or inducing oncogene expression [69]. Epigenetic modifications are reversible changes that affect gene expression but cause no change to the DNA sequence. They include histone modifications and DNA methylation among others. DNA methylation is linked to gene silencing and hyper-methylation of various tumor suppressor promoter regions is common in cancers [70]. Histone methylation by the Polycomb Repressive Complex 2 (PRC2) is a major mechanism of gene silencing [71]. EZH2 is the methyltransferase in the PRC2 complex, responsible for adding three methyl groups onto lysine 27 of histone 3 (H3K27me3) [71]. Elevated expression of EZH2 is seen in breast cancers and has been linked to poor prognosis [72]. Overexpressing EZH2 under the control of the MMTV promoter (MMTV-EZH2) resulted in the formation of mammary gland hyperplasia and adenomas, the first stage in breast cancer progression [73]. This study highlighted that EZH2 may be important for tumor initiation and the progression of normal epithelium to hyperplasia [73]. Gene expression and DNA methylation status were assessed in glands of MMTV-PyMT animals at various stages of disease progression [74, 75]. Analysis revealed an increase in differentially expressed genes, as well as differentially methylated cytosines as the disease progresses [74, 75]. Interestingly, hypermethylation of promoters, which was associated with reduced gene expression, were enriched for in the late stage carcinomas compared to earlier hyperplasia [74]. This was also coupled to an increased expression of PRC components including EZH2 [74]. These studies highlight that several epigenetic players, DNA and histone methylations, are associated with tumor progression and may act in synergy to suppress expression of tumor suppressors. Functionally, conditional ablation of EZH2 in the MTB/tetO-MIC model significantly delayed tumor onset and decreased the incidence of lung metastasis, showing that EZH2 is essential for progression of mammary tumors and metastasis [42]. This phenotype was also recapitulated through pharmacological inhibition of EZH2 [42]. Off note, treatment of an ErbB2 driven mouse model with an inhibitor of EZH2 revealed strong synergy with anti-ErbB2 targeted therapy which highlights the importance of EZH2 in several cancers and emphasizes its clinical relevance [76].

## The genetic background of MMTV-PyMT influences tumor onset and metastasis

Mouse genetic background plays a fundamental role in the biology of the tumor. FVB/NJ MMTV-PyMT tumors arise significantly faster than in C57Bl/6J MMTV-PyMT [77]. Furthermore, identical knockouts in these different backgrounds give rise to different results. Prominently, iNOS knockout only shows an effect on tumor onset and metastasis in the C57Bl/6J but not the FVB/NJ background [77]. A similar effect was shown when RAG1 was knocked out in both FVB/NJ and C57Bl/6J, with only the C57Bl/6J strain showing accelerated onset [10, 43]. Interestingly, MMTV-PyMT FVB/NJ was crossed one generation into 27 inbred mouse background strains, 13 of which displayed a significant reduction in pulmonary metastasis and 10 had altered tumor kinetics compared to the parental FVB/NJ background [78]. This study supported the role of metastatic modifier genes, potential tumor suppressors or proto-oncogenes, that play an important role in tumorigenesis and metastasis [78, 79]. Polymorphisms that affect gene expressions or silent mutations in genes that display slightly altered activity levels during neoplasia in these inbred strains are sufficient to alter metastatic burden. This could at least in part explain the biological differences between the FVB/NJ and C57Bl/6J backgrounds discussed above. Such loci potentially play a role in generating inter-patient tumor heterogeneity and might allow identification of metastasis susceptible patients (metastasis-predictive gene expression signatures), even from premalignant, normal tissue, and infiltrating non-neoplastic cells [79]. *Mtes1* is one metastatic modifier locus identified to be different between the inbred strains [80]. Particularly within the *Mtes1* locus the *Sipa1* gene, which encodes a GTPase activated protein, harbored a threonine to alanine substitution in the lower metastatic strain, which increased SIPA1 binding to its negative regulator, AQP2 [81]. Further support of the relevance of this GEMM was that SIPA1 expression is associated with metastatic progression of human prostate cancer [81]. It is worth noting that MMTV-PyMT FVB/NJ × AKR/J is a highly metastatic strain, whereas MMTV-PyMT FVB/NJ × DBA/2J is a low metastatic strain which are associated with the polymorphisms of *Sipa1* [78, 79].

## The role of the host tumor microenvironment in mammary tumorigenesis: Implications for disease progression and metastasis

### Mammary gland density

In addition to providing genetic and mechanistic insight into tumor initiation, PyMT driven mouse models have also been used to identify and study various risk factors for developing breast cancers. For several decades, breast tissue density has been linked to breast cancer incidence in women [82, 83]. While the evidence was mostly correlational, it was observed that higher breast density is associated with higher breast cancer risk and that hyperplasia and ductal carcinomas in situ often begin in the densest parts of the breast [84]. The first causal link of breast density to cancer incidence came using the MMTV-PyMT mouse carrying a mutation that makes collagen resistant to degradation by collagenases (Col1a1^tm1jae^) [85]. This mutation resulted in increased collagen deposition in the breast, mimicking high-density human breast tissue [85]. The increased tissue density was the stimulating event driving tumor formation, increasing the number of tumors per mouse, as well as number of metastatic lesions [85]. This was the first evidence linking breast tissue density to breast cancer incidence and verifying it as a major risk factor for breast cancer development. Further analysis found this increased tumorigenesis to be mediated by COX2, raising the possibility of COX2 inhibitors as therapeutic options for breast cancer patients with high breast tissue density [86].

### Macrophages

Macrophages are one of, if not, the most abundant immune cells in the tumor microenvironment [87]. As tumors grow in MMTV-PyMT mice, a decrease in the mammary tissue macrophages (MTMs) (MHCII^hi^, CD11b^hi^) coupled with an increase in tumor associated macrophages (TAMs) (CD11b^lo^, MHCII^hi^, F4/80+, CD64+, MerTK+) is observed [87–89]. Macrophages promote nearly all the stages of cancer metastasis such as preconditioning the pre-metastatic niche, supporting tumor invasion, cell extravasation and colonization of the secondary organ [89]. On the extreme scale, macrophages can be polarized to either classically activated macrophages (M1), which is associated with response to interferon gamma and lipopolysaccharides [87, 89]. These macrophages are associated with production of pro-inflammatory cytokines and antigen presentation [87–89]. This population would be associated with improved prognosis. On the other end of the scale, macrophages can be alternatively activated (M2), which are associated with anti-inflammatory cytokines and promote tissue remodeling, supporting cancer progression and metastasis [87–89]. Despite popular belief, data arising from characterization of TAMs in MMTV-PyMT revealed that they are not strictly M2-polarized [88]. Mammary tissue macrophages in untransformed glands, MTMs, resembled M2 macrophages more than TAMs [88], which would be consistent with their role of tissue remodeling in a very dynamic organ such as the murine mammary gland. Franklin et al. proposed a model in which CCR2+ monocytes develop into TAMs, with Vcam1+ expressing cells being mature TAMs in MMTV-PyMT [87, 88]. Nonetheless, microarray analysis of transcripts expressed in phagocytic myeloid cells in end stage MMTV-PyMT tumors reveal expression of genes associated with mediating immune responses and angiogenesis [90]. Presence of Vcam1+ TAMs was correlated with an increased number of exhausted T cells (PD1+Gzmb−CD8)+, highlighting the crosstalk between the innate and adaptive immune responses in modulation of the tumor immune response [88].

The role of macrophages in tumor progression and metastasis was highlighted using a null mutant of CSF1, a macrophage growth factor [11]. Homozygous null CSF1 MMTV-PyMT mice exhibited a remarkable decrease in tumor progression to advanced disease and lacked evidence of pulmonary metastasis [11]. This was associated with a dramatic decrease in F4/80+ macrophages in the primary tumor [11]. This phenotype was rescued using an overexpression CSF1 animal [11]. Furthermore, overexpression of CSF1 in heterozygous null CSF1 MMTV-PyMT animals accelerated progression to advanced disease and accelerated the emergence of pulmonary metastasis [11]. Again, this was coinciding with an elevated number of F4/80 cells in the primary tumors [11]. The delayed progression in CSF1 null animals was also rescued by epithelial overexpression of VEGF [91], suggesting that macrophages may induce VEGF expression in PyMT cells.

Using an in vivo migration assay and multiphoton imaging of migratory cells revealed a paracrine loop that exists between PyMT carcinoma cells and macrophages [9]. Macrophages enhance cell migration via expression of epidermal growth factor (EGF) which binds the EGF receptor found specifically on the cancer cells [9]. In turn, PyMT cells secrete CSF1, which binds the CSF1 receptor on macrophages promoting their taxis towards the tumor cells [9]. Imaging revealed that migratory cells leaving the primary tumor were almost exclusively PyMT carcinoma cells and macrophages [9]. Depletion of macrophages, using the CSF1 knockout, reduced the number of migratory cells [9]. Another study knocked out S1P receptors specifically in TAMs using an F4/80-Cre strain, revealed defects in lymphangiogenesis and corroborated a reduced pulmonary metastatic burden [92]. This data argues that PyMT not only metastasize via the hematogenous route (see angiogenesis section) but also via the lymphatics.

### T-lymphocytes

There is also compelling evidence supporting the role of adaptive immune cells, such as T cells, during PyMT tumor progression. The number of CD4+ T cells along with F4/80+ macrophages increases during progression [10]. Depletion of CD4+ T cells was associated with reduced number of circulating tumor cells and pulmonary metastasis [10]. Furthermore, CD4+ deficient lesions expressed elevated levels of M1 macrophage cytokines (TNFα and Nos2) [10]. Further analysis revealed that IL-4 released by CD4+ T cells was capable of polarizing macrophages into more M2-like state while suppressing the M1-like phenotype and promoting macrophage expression of EGF, which establishes the paracrine loop between tumor cells and macrophages [10, 10, 87, 89].

The role of T cells is not limited to the primary site, but is also implicated at the metastatic niche. PyMT tumors overexpressing macrophage stimulating protein (PyMT-MSP) show more local invasive behavior and lead to the development of osteolytic bone metastasis, not seen in the parental PyMT mice [93]. This highlights the versatility of PyMT models, as additional oncogenes may be overexpressed or knocked-in (or tumor-suppressor knockouts) that promote metastatic-organ tropism. Generation of such models is important to allow development of therapeutics that appropriately target metastatic disease, which is not limited to lungs.

MSP binds its receptor, Ron (MST1R), which is expressed on a variety of cells including breast cancer cells [94]. A synergistic transplant model of PyMT-MSP cells in animals harboring mutant Ron receptors lacking tyrosine kinase ability (Ron TK−/−), demonstrated that the effect is driven by the host environment [95]. PyMT-MSP harboring Ron TK−/− animals had elevated splenic CD8+ T cells and elevated CD8+ cells in the tumor margin. CD8+ cells also infiltrated the metastatic lung and expressed more TNFα [95]. CD8+ cells from Ron TK−/− tumors had improved cytotoxic ability [95]. Antibody depletion of CD8+ cells or the use of a SCID mouse rescued the metastatic defect of Ron TK−/− animals [95]. Consistent with this, pharmacological inhibition of Ron prevented formation of overt metastasis in the lung post metastatic colonization [95]. Further studies have also revealed that suppression of the MSP-RON axis potentiates the effect of anti-CTLA4 checkpoint blockade via crosstalk between macrophages and cytotoxic T cells in MMTV-PyMT primary tumors, as well as at the metastatic lung [96].

Overall, these studies support the presence of crosstalk between the innate and adaptive immune system that contributes to metastasis. Furthermore, it raises the possibility of targeting the immune microenvironment at the metastatic site, which would be important in improving therapeutic regimens for patients. Studies such as these are possible due to the high penetrance of metastasis of the PyMT models within an immunocompetent background.

### Neutrophils

Another subset of immune cells that play a role during cancer progression and metastasis are the neutrophils. In the pre-metastatic lung of MMTV-PyMT mice, an influx of neutrophils (CD11b+Ly6G+) occurs and their numbers continue to increase during the metastatic progression [97]. Animals with depleted neutrophils display a reduction in spontaneous pulmonary metastasis [97]. Moreover, the CD11b+LyG^hi^ cell population had elevated expression of extracellular matrix chondroitin proteoglycan, versican, which promotes growth of the metastatic lesions and mesenchymal to epithelial transition which is required for development of the metastasis [97].

### Natural killers

Natural killer (NK) cells also play an important role in immune surveillance [98]. Genetic and antibody depletion of natural killer cells increased pulmonary metastatic burden in PyMT animals [98]. Intranasal IL-12 treatment to promote activation of NK cells, reduced pulmonary metastasis, emphasizing their role in the metastatic niche and not just in the primary tumor [98].

### Angiogenesis

**Fig. 4:**
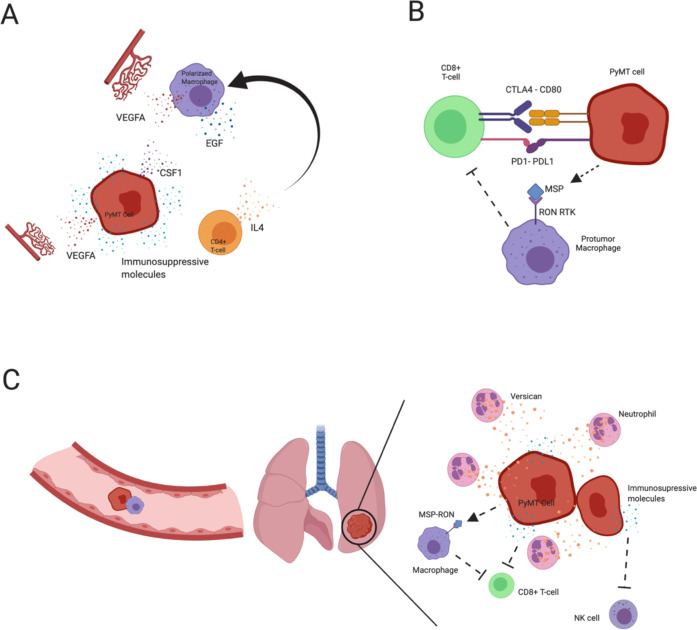
TME factors that promote metastasis in PyMT. **a** At the primary tumor, PyMT cancer cells secrete a range of immunosuppressive and chemotactic molecules. CD4+ T cells recruited to the primary tumor secrete IL-4 which polarizes macrophages to the M2 state. Polarized macrophages in turn release EGF which promotes cancer cell growth and migration. Myeloid and cancer cell derived VEGFA 6 promotes angiogenesis. PyMT cancer cells release CSF1 which promote taxis of macrophages. **b** Within the primary tumor, PyMT cancer cells suppress activity of cytotoxic T cells both directly via PD1/PDL1 and CTLA4/CD80 interactions, inhibiting their tumor killing ability. PyMT also suppress cytotoxic T cell activity indirectly via release of MSP which binds the RON receptor on macrophages which leads to suppression of cytotoxic T cell activity. **c** PyMT cancer cells metastasize via hematogenous route along with macrophages to reach the lung. At the metastatic site, neutrophils release versican which supports growth of the PyMT cancer cells and promotes mesenchymal to epithelial transition. Within the lung microenvironment, PyMT cancer cells supress the cytotoxic T cells and natural killer (NK) cells directly and indirectly. Created with Biorender.com .

Another key tumor microenvironmental cue is the process of angiogenesis. As lesions of MMTV-PyMT progress to early carcinomas, they are associated with increased vascular density [99]. As the tumors progress to malignancy, the length of the vessels and their tortuosity increases dramatically [99]. Myeloid derived macrophages are key for PyMT progression and metastasis (discussed previously) and are a major source of VEGF [87–89]. However, knockout of VEGF in the myeloid compartment through the LysM-Cre strain led to faster tumor progression to malignancy compared to their wildtype counterparts [99]. This interesting phenomenon might be explained, at least partially, by the time of anti-VEGF treatment/ablation. Mice harboring LysM-Cre/VEGF conditional are depleted of macrophage derived VEGF early on during tumor initiation, potentially allowing for adaptation mechanism to come into play. Another study neutralized VEGF receptor 2 by DC101 and noted a reduction in growth [100]. Analysis of the immune microenvironment of these tumors revealed a significant increase in IFN γ producing cytotoxic T cells [100]. However, this was associated with an increase in PDL-1 expressing PyMT tumor cells as a negative feedback mechanism [100]. It is therefore reasonable to suggest that VEGF knockout in macrophages results in elevated expression of tumoral PDL-1 during initiation, which leads to immune evasion and supports the faster growing tumors. Indeed, co-treatment of PyMT tumors with both DC101 and anti-PDL-1 was sufficient to restrict tumor growth and was associated with vasculature normalization [100]. Furthermore, neutralization of angiopoietin 2, a secreted endothelial cell growth factor, by a monoclonal antibody (3.19.3) impeded tumor growth in MMTV-PyMT mice at both early and late stages of progression [101]. This neutralization led to vascular regression and increased pericyte coverage of the vessels, increased tumor hypoxia, necrosis and enhanced recruitment of MRC1+(CD206) tumor associated macrophages [101]. Enhanced recruitment of macrophages was also noted post treatment using combretastatin A4 phosphate, a vascular disrupting agent, suggesting it as a general mechanism in attempt to rescue the tumor vasculature after therapy [102]. Interestingly, tumors treated with 3.19.3 did not display rebound angiogenesis despite elevated levels of VEGFa and MRC1+ macrophage infiltrate, highlighting a key role that angiopoietin 2 may play in promoting vascularization in PyMT driven tumors [101]. Future application of this monoclonal therapeutic approach may have important implications on breast cancer treatments. The mechanisms by which PyMT cells model the microenvironment and suppress the anti-cancer immune response to promote metastasis are summarized in Fig. 4.

Despite the focus on the hematogenous route of metastasis, the PyMT model does metastasize, albeit to a low extent, to the lymph node, a common metastatic site in human breast cancer [103]. Lymph node metastasis was significantly increased in animals lacking expression of the cytochrome P450 Cyp2c44, similar to MMP8 suggesting its inhibition is inadvisable [103].

## PyMT GEMMS as a preclinical platform for therapeutic testing

In addition to providing valuable genetic and mechanistic insights into breast tumorigenesis, PyMT-driven GEMMS have been a cornerstone for pre-clinical development and testing of potential therapies. From chemotherapies to targeted therapies, immunotherapies and cancer vaccines, all have been tested for their efficiency and dosage using PyMT mouse models. Table 2 highlights some of the therapies tested in PyMT models grouped based on the drug type. It is important to note that several of these therapeutics have also been tested in combination with other treatments, chemotherapies and radiation similar to what physicians prescribe their patients following breast cancer diagnosis. Thus, PyMT models are clinically relevant mouse models used for pre-clinical testing of various therapeutic methods to determine doses, safety, and efficacy.

**Table 2:** Therapeutics with clinical potential tested in PyMT.

Drug name	Target/mechanism of action	Effect	Reference
Immunotherapies			
ANTI-PDL1	Blocks the Programmed death ligand (PDL1) that is commonly expressed by tumor cells to inhibit T cell activity.	Increased CD8+ T cell activity, decreased tumor burden.	[100]
ADIL12– B7-1	Adenovirus expressing the cytokine IL12 and the co-stimulatory molecule B7-1 that activates T cells.	Complete regression of tumors with no detected relapse. At lower dose, it delayed tumor growth and was still effective.	[110]
ANTI-IL4	Monoclonal antibody against the secreted cytokine IL4. Antibody results in increase M1 macrophages polarization.	No change in tumor onset, latency or burden. But significantly decreased the number of lung metastases.	[90]
Drugs targeting DNA or RNA			
EZN-3920	ErbB3 antisense	Significant reduction in tumor growth and inhibits tumor progression.	[66]
Targeted therapies			
GSK-126	Ezh2 methyltransferase inhibitor	Decrease in hyperplasias and delay in tumor onset. Complete block of metastasis	[42]
Buthionine-[S, R]-sulfoximine (BSO)	Inhibitor of GSH (Glutathione) synthesis.	Early treatment increased ROS levels, reduced tumor burden and disrupted progression to later stages. Treatment upon tumor onset did not change ROS levels and had no effect on tumor burden, suggesting a reason why clinical trials have failed.	[108]
Anti-MMR nanobody	Nanobody targeting CD206+ TAMs	Specially binds to and label TAMs, allowing for imaging of tumor stroma and hypoxic regions.	[111]
Anti-ANG2 antibody (3.19.3)	Blocks ANG2’s ability to bind Tie2, inhibiting an important pro-angiogenic axis	Interfered with angiogenesis, disrupted blood vasculature and inhibits tumor progression	[101]
ANTI-CSF1	Inhibits colony stimulating factor, a macrophage differentiation factor.	Depleted macrophages and delayed tumor regrowth following radiation	[112]
PLX3397	Competitive ATP inhibitor for CSF1 receptor	Eliminated tumor associated macrophages and delayed tumor growth after radiation	[112]
DC101 antibody	anti-VEGF	Reduced tumor growth due to impairment of angiogenesis. It also increased the levels of PD-L1, sensitizing tumors for combination with anti-PDL1 antibody. This combination led to significant reduction in tumor burden.	[100]
Lapatinib	Tyrosine kinase inhibitor (ErbB2 and EGFR)	Delay tumor growth,	[66]
Tamoxifen	Selective estrogen receptor modulator (SERM) that inhibits ER signaling.	Inhibited tumor growth and decreased tumor volume.	[63]
Chemotherapy and radiotherapy			
5 GY focal gamma irradiation from cesium source	Ionizing radiation	Tumor regressed, but regrowth occurs after a period of stasis.	[112]
Doxorubicin	Chemotherapy. Inhibits topoisomerase 2, an enzyme important for DNA replication.	Reduced tumor volume due to necrotic cancer cell death.	[113]

## Conclusions

Overall, we highlighted several key studies that made use of either PyMT GEMM that not only provided insight into how the model functions and behaves, but also provided insight into clinically relevant cancer biology. Many findings from the PyMT models are directly applicable to those of human cancers supporting that these models are clinically relevant. Despite the rising popularity of human xenografts, whether of established cell lines or from a patient, these models make use of immune compromised mice, which are unable to mount an adaptive immune response [104]. This makes pre-clinical testing of drugs that make use of the adaptive immune system impossible. Moreover, in these models, human cells are growing in a murine microenvironment which raises the issue of species incompatibilities and a foreign microenvironment [104]. Co-transplant of human stroma (fibroblasts) has also become popular but they do not address the issue of metastasis as the seeded metastasis is now associated with murine stroma [104]. In addition, xenografts cannot be used to assess tumor onset as they come from established tumor lines and their implantation into the fat pad does not accurately represent tumor progression as there is no myoepithelial layer surrounding the luminal cells. Furthermore, genetic manipulation of the tumor as a whole is currently not feasible. Therefore, the use of PyMT derived GEMMs to model breast cancer remains just as valuable as they ever were.

## References

[1] Brenner DR, Weir HK, Demers AA, Ellison LF, Louzado C, Shaw A (2020). Projected estimates of cancer in Canada in 2020. CMAJ.

[2] Guy CT, Cardiff RD, Muller WJ (1992). Induction of mammary tumors by expression of polyomavirus middle T oncogene: a transgenic mouse model for metastatic disease. Mol Cell Biol.

[3] Lin EY, Jones JG, Li P, Zhu L, Whitney KD, Muller WJ (2003). Progression to malignancy in the polyoma middle T oncoprotein mouse breast cancer model provides a reliable model for human diseases. Am J Pathol.

[4] Gross L (1953). A filterable agent, recovered from Ak leukemic extracts, causing salivary gland carcinomas in C3H mice. Proc Soc Exp Biol Med.

[5] Treisman R, Novak U, Favaloro J, Kamen R (1981). Transformation of rat cells by an altered polyoma virus genome expressing only the middle-T protein. Nature.

[6] Pfefferle AD, Herschkowitz JI, Usary J, Harrell JC, Spike BT, Adams JR (2013). Transcriptomic classification of genetically engineered mouse models of breast cancer identifies human subtype counterparts. Genome Biol.

[7] Lapidus R, Nass S, Davidson N (1998). The loss of estrogen and progesterone receptor gene expression in human breast cancer. J Mammary Gland Biol Neoplasia.

[8] Lopez-Tarruella S, Schiff R (2007). The dynamics of estrogen receptor status in breast cancer: re-shaping the paradigm. Clin Cancer Res.

[9] Wyckoff JB, Wang Y, Lin EY, Li J, Goswami S, Stanley ER (2007). Direct visualization of macrophage-assisted tumor cell intravasation in mammary tumors. Cancer Res.

[10] Denardo DG, Barreto JB, Andreu P, Vasquez L, Tawfik D, Kolhatkar N (2009). CD4+ T cells regulate pulmonary metastasis of mammary carcinomas by enhancing protumour properties of macrophages. Cancer Cell.

[11] Lin EY, Nguyen AV, Russell RG, Pollard JW (2001). Colony-stimulating factor 1 promotes progression of mammary tumors to malignancy. J Exp Med.

[12] Maglione J, Moghanaki D, Young LJ, Manner CK, Ellies LG, Joseph SO (2001). Transgenic polyoma middle-T mice model premalignant mammary disease. Cancer Res.

[13] Ben-David U, Ha G, Khadka P, Jin X, Wong B, Franke L (2016). The landscape of chromosomal aberrations in breast cancer mouse models reveals driver-specific routes to tumorigenesis. Nat Commun.

[14] Ross C, Szczepanek K, Lee M, Yang H, Qiu T, Sanford JD (2020). The genomic landscape of metastasis in treatment-naïve breast cancer models. PLoS Genet.

[15] Montagna C, Lyu M, Hunter K, Lukes L, Lowther W, Reppert T (2003). The Septin 9 (MSF) Gene is amplified and overexpressed in mouse mammary adenocarcinomas and human breast cancer cell lines. Cancer Res.

[16] Hodgson JG, Malek T, Bornstein S, Hariono S, Ginzinger DG, Muller WJ (2005). Copy number aberrations in mouse breast tumors reveal loci and genes important in tumorigenic receptor tyrosine kinase signalling. Cancer Res.

[17] Rennhack J, To B, Wermuth H, Andrechek ER (2017). Mouse models of breast cancer share amplification and deletion events with human breast cancer. J Mammary Gland Biol Neoplasia.

[18] Orsetti B, Nugoli M, Cervera N, Chuchana P, Ursule L, Redon R (2004). Genomic and expression profiling of chromosome 17 in breast cancer reveals complex patterns of alterations and novel can- didate genes. Cancer Res.

[19] Rennhack JP, To B, Swiatnicki M, Dulak C, Ogrodizinski MP, Zhang Y (2019). Integrated analyses of murine breast cancer models reveal critical parallels with human disease. Nat Commun.

[20] Swiatnicki MR, Rennhack JP, Andrecheck ER (2020). PTPRH Mutations in NSCLC regulates EGFR phosphorylation. J Thorac Oncol.

[21] Xiao B, Zuo D, Hirukawa A, Cardiff RD, Lamb R, Sonenberg N (2020). Rheb1-Independent Activation of mTORC1 in Mammary Tumors Occurs through Activating Mutations in mTOR. Cell Rep.

[22] Cai Y, Nogales-Cadenas R, Zhang Q, Lin J, Zhang W, O’Brien K, et al. Transcriptomic dynamics of breast cancer progression in the MMTV-PyMT mouse model. 2017;18:1–14. 10.1186/s12864-017-3563-3 PMC5316186 28212608

[23] Dunant NM, Senften M, Ballmer-Hofer K (1996). Polyomavirus middle-T antigen associates with the kinase domain of Src-related tyrosine kinases. J Virol.

[24] Campbell KS, Ogris E, Burke B, Su W, Auger KR, Druker BJ (1994). Polyoma middle tumor antigen interacts with SHC protein via the NPTY (Asn-Pro-Thr-Tyr) motif in middle tumor antigen. Proc Natl Acad Sci USA.

[25] Whitman M, Kaplan DR, Schaffhausen B, Cantley L, Roberts TM (1985). Association of phosphatidylinositol kinase with polyoma middle-T competent for transformation. Nature.

[26] Su W, Liu W, Schaffhausen BS, Roberts TM (1995). Association of polyomavirus middle T with phospholipase C-gamma1. J Biol Chem.

[27] Pallas DC, Shahrik LK, Martin BL, Jaspers S, Miller TB, Brautigan DL (1990). Polyoma small and middle T antigens and SV40 small t antigen form stable complexes with protein phosphatase 2A. Cell.

[28] Hunter T, Hutchinson MA, Eckhart W (1984). Polyoma middle-T antigen can be phosphorylated on tyrosine at multiple sites in vitro. EMBO J.

[29] Ong SH, Dilworth S, Hauck-Schmalenberger I, Pawson T, Kiefer F (2001). ShcA and Grb2 mediate polyoma middle T antigen-induced endothelial transformation and Gab1 tyrosine phosphorylation. EMBO J.

[30] Kaplan DR, Whitman M, Schaffhausen B, Raptis L, Garcea RL, Pallas D (1986). Phosphatidylinositol metabolism and polyoma-mediated transformation. PNAS.

[31] Guy CT, Muthuswamy SK, Cardiff RD, Soriano P, Muller WJ (1994). Activation of the c-Src tyrosine kinase is required for the induction of mammary tumors in transgenic mice. Genes Dev.

[32] Kim H, Laing M, Muller W (2005). c-Src-null mice exhibit defects in normal mammary gland development and ERalpha signaling. Oncogene.

[33] Marcotte R, Smith HW, Sanguin-Gendreau V, McDonough RV, Muller WJ (2012). Mammary epithelial-specific disruption of c-Src impairs cell cycle progression and tumorigenesis. Proc Natl Acad Sci USA.

[34] Webster MA, Hutchinson JN, Rauh MJ, Muthuswamy SK, Anton M, Tortorice CG (1998). Requirement for both Sch and phosphatidylinositol 39 kinase pathways in polyomavirus middle T-mediated mammary tumorigenesis. Mol Cell Biol.

[35] Ursini-Siegel J, Hardy WR, Zuo D, Lam SH, Sanguin-Gendreau V, Cardiff RD (2008). ShcA signalling is essential for tumour progression in mouse models of human breast cancer. EMBO J.

[36] Ahn R, Sabourin V, Bolt AM, Hebert S, Totten S, De Jay N (2017). The Shc1 adaptor simultaneously balances Stat1 and Stat3 activity to promote breast cancer immune suppression. Nat Commun.

[37] Wagner K, Wall RJ, St-Onge L, Gruss P, Wynshaw-Boris A, Garrett L (1997). Cre-mediated gene deletion in the mammary gland. Nucleic Acids Res.

[38] White DE, Kurpios NA, Zuo D, Hassell JA, Blaess S, Mueller U (2004). Targeted disruption of beta1-integrin in a transgenic mouse model of human breast cancer reveals an essential role in mammary tumor induction. Cancer Cell.

[39] Rao T, Ranger JJ, Smith HW, Lam SH, Chodosh L, Muller WJ (2014). Inducible and coupled expression of the polyomavirus middle T antigen and Cre recombinase in transgenic mice: an in vivo model for synthetic viability in mammary tumour progression. Breast Cancer Res.

[40] Gunther EJ, Belka GK, Wertheim GB, Wang J, Hartman JL, Boxer RB (2002). A novel doxycycline-inducible system for the transgenic analysis of mammary gland biology. FASEB J.

[41] Jones LM, Broz ML, Ranger JJ, Ozcelik J, Ahn R, Zuo D (2016). STAT3 Establishes an immunosuppressive microenvironment during the early stages of breast carcinogenesis to promote tumor growth and metastasis. Cancer Res.

[42] Hirukawa A, Smith HW, Zuo D, Dufour CR, Savage P, Bertos N (2018). Targeting EZH2 reactivates a breast cancer subtype-specific anti-metastatic transcriptional program. Nat Commun.

[43] Gross ET, Han S, Vemu P, Peinado CD, Marsala M, Ellies LG (2017). Immunosurveillance and immunoediting in MMTV-PyMT-induced mammary oncogenesis. Oncoimmunology.

[44] Qin J, Yan L, Zhang J, Zhang WD. STAT3 as a potential therapeutic target in triple negative breast cancer: a systematic review. J Exp Clin Cancer Res. 2019;38. 10.1186/s13046-019-1206-z 10.1186/s13046-019-1206-z PMC6518732 31088482

[45] Halaoui R, Rejon C, Chatterjee SJ, Szymborski J, Meterissian S, Muller WJ (2017). Progressive polarity loss and luminal collapse disrupt tissue organization in carcinoma. Genes Dev.

[46] Allred DC, Mohsin SK, Fuqua SA (2001). Histological and biological evolution of human premalignant breast disease. Endocr Relat Cancer.

[47] Simond AM, Ling C, Moore MJ, Condotta SA, Richer MJ, Muller WJ (2020). Point-activated ESR1(Y541S) has a dramatic effect on the development of sexually dimorphic organs. Genes Dev.

[48] Kleiner D, Stetler-Stevenson W (1999). Matrix metalloproteinases and metastasis. Cancer Chemother Pharm.

[49] Winer A, Adams S, Mignatti P (2018). Matrix Metalloproteinase Inhibitors in Cancer Therapy: Turning Past Failures Into Future Successes. Mol Cancer Therapeutics.

[50] Decock J, Hendrickx W, Thirkettle S, Gutiérrez-Fernández A, Robinson SD, Edwards DR (2015). Pleiotropic functions of the tumor- and metastasis-suppressing matrix metalloproteinase-8 in mammary cancer in MMTV-PyMT transgenic mice. Breast Cancer Res.

[51] Höckel M, Vaupel P (2001). Tumor hypoxia: definitions and current clinical, biologic, and molecular aspects. J Natl Cancer Inst.

[52] Majumdar AJ, Wong WJ, Simon MC (2010). Hypoxia-inducible factors and the response to hypoxic stress. Mol Cell.

[53] Jin M, Lee H, Park IA, Chung YR, Im SA, Lee KH (2016). Overexpression of HIF1α and CAXI predicts poor outcome in early-stage triple negative breast cancer. Virchows Arch.

[54] Schwab LP, Peacock DL, Majumdar D, Ingels JF, Jensen LC, Smith KD (2012). Hypoxia-inducible factor 1α promotes primary tumor growth and tumor-initiating cell activity in breast cancer. Breast Cancer Res.

[55] Liao D, Corle C, Seagroves TN, Johnson RS (2007). Hypoxia-inducible factor -1α is a key regulator of metastasis in a transgenic model of cancer initiation and progression. Cancer Res.

[56] Creighton CJ, Li X, Landis M, Dixon JM, Neumeister VM, Sjolund A (2009). Residual breast cancers after conventional therapy display mesenchymal as well as tumor-initiating features. Proc Natl Acad Sci USA.

[57] Ma J, Lanza DG, Guest I, Uk-Lim C, Glinskii A, Glinsky G, et al. Characterization of mammary cancer stem cells in the MMTV-PyMT mouse model. Tumor Biol. 2012; 10.1007/s13277-012-0458-4 10.1007/s13277-012-0458-4 22878936

[58] Asselin-Labat ML, Sutherland KD, Vaillant F, Gyorki DE, Wu D, Holroyd S (2011). Gata-3 negatively regulates the tumor-initiating capacity of mammary luminal progenitor cells and targets the putative tumor suppressor caspase-14. Mol Cell Biol.

[59] Ni T, Li X, Lu N, An T, Liu Z, Fu R (2016). Snail1-dependent p53 repression regulates expansion and activity of tumour-initiating cells in breast cancer. Nat Cell Biol.

[60] Dadi S, Chhangawala S, Whitlock BM, Franklin RA, Luo CT, Oh SA (2016). Cancer Immunosurveillance by Tissue-Resident Innate Lymphoid Cells and Innate-like T Cells. Cell.

[61] Boyle ST, Faulkner JW, McColl SR, Kochetkova M (2015). The chemokine receptor CCR6 facilitates the onset of mammary neoplasia in the MMTV-PyMT mouse model via recruitment of tumor-promoting macrophages. Mol Cancer.

[62] Harbeck N, Penault-Llorca F, Cortes J, Gnant M, Houssami N, Poortmans P (2019). Breast cancer. Nat Rev Dis Prim.

[63] Butt SA, Sogaard LV, Ardenkjaer-Larsen JH, Lauritzen MH, Engelholm LH, Paulson OB (2015). Monitoring mammary tumor progression and effect of tamoxifen treatment in MMTV-PyMT using MRI and magnetic resonance spectroscopy with hyperpolarized [1-^13^C] Pyruvate. Magn Reson Med.

[64] Qin L, Liao L, Redmond A, Young L, Yuan Y, Chen H (2008). The AIB1 oncogene promotes breast cancer metastasis by activation of PEA3-mediated matrix metalloproteinase 2 (MMP2) and MMP9 expression. Mol Cell Biol.

[65] Dabrosin C, Palmer K, Muller WJ, Gauldie J (2003). Estradiol promotes growth and angiogenesis in polyoma middle T transgenic mouse mammary tumor explants. Breast Cancer Res Treat.

[66] Cook RS, Garrett JT, Sanchez V, Stanford JC, Young C, Chakrabarty A (2011). ErbB3 ablation impairs PI3K/Akt-dependent mammary tumorigenesis. Cancer Res.

[67] Jang I, Beningo KA (2019). Integrins, CAFs and mechanical forces in the progression of cancer. Cancers.

[68] Lahlou H, Sanguin-Gendreau V, Zuo D, Cardiff RD, McLean GW, Frame MC (2007). Mammary epithelial-specific disruption of the focal adhesion kinase blocks mammary tumor progression. Proc Natl Acad Sci USA.

[69] Gronbaek K, Hother C, Jones PA (2007). Epigenetic changes in cancer. APMIS.

[70] Esteller M (2002). CpG island hypermethylation and tumor suppressor genes: a booming present, a brighter future. Oncogene.

[71] Di Croce L, Helin K (2013). Transcriptional regulation by Polycomb group proteins. Nat Struct Mol Biol.

[72] Kleer CG, Cao Q, Varambally S, Shen R, Ota I, Tomlins SA (2003). EZH2 is a marker of aggressive breast cancer and promotes neoplastic transformation of breast epithelial cells. Proc Natl Acad Sci USA.

[73] Li X, Gonzalez ME, Toy K, Filzen T, Merajver SD, Kleer CG (2009). Targeted overexpression of EZH2 in mammary gland disrupts ductal morphoenesis and causes epithelial hyperplasia. Am J Pathol.

[74] Cai Y, Nogales-Cadenas R, Zhang Q, Lin JR, Zhang W, O’Brien K (2017). Transcriptomic dynamics of breast cancer progression in the MMTV-PyMT mouse model. BMC Genomics.

[75] Cai Y, Lin JR, Zhang Q, O’Brien K, Montagna C, Zhang ZD (2018). Epigenetic alterations to Polycomb targets precede malignant transition in a mouse model of breast cancer. Sci Rep.

[76] Hirukawa A, Singh S, Wang J, Rennhack JP, Swiatnicki M, Sanguin-Gendreau V (2019). Reduction of global H3K27me(3) enhances HER2/ErbB2 targeted therapy. Cell Rep.

[77] Davie SA, Maglione JE, Manner CK, Young D, Cardiff RD, MacLeod CL (2007). Effects of FVB/NJ and C57Bl/6J strain backgrounds on mammary tumor phenotype in inducible nitric oxide synthase deficient mice. Transgenic Res.

[78] Lifsted T, Le Voyer T, Williams M, Muller W, Klein-Szanto A, Buetow KH (1998). Identification of inbred mouse strains harboring genetic modifiers of mammary tumor age of onset and metastatic progression. Int J Cancer.

[79] Lukes L, Crawford NP, Walker R, Hunter KW (2009). The origins of breast cancer prognostic gene expression profiles. Cancer Res.

[80] Hunter KW, Broman KW, Le Voyer T, Lukes L, Cozma D, Debies MT (2001). Predisposition to efficient mammary tumor metastatic progression is linked to the breast cancer metastasis suppressor gene *Brms1*. Cancer Res.

[81] Park YG, Zhao X, Lesueur F, Lowy DR, Lancaster M, Pharoah P (2005). Sipa1 is a candidate for underlying the metastasis efficiency modifier locus Mtes1. Nat Genet.

[82] Boyd NF, Lockwood GA, Byng JW, Tritchler DL, Yaffe MJ (1998). Mammographic densities and breast cancer risk. Cancer Epidemiol Biomark Prev.

[83] Martin LJ, Melnichouk O, Guo H, Chiarelli AM, Hislop TG, Yaffe MJ (2010). Family history, mammographic density, and risk of breast cancer. Cancer Epidemiol Biomark Prev.

[84] Gill JK, Maskarinec G, Pagano I, Kolonel LN (2006). The association of mammographic density with ductal carcinoma in situ of the breast: the Multiethnic Cohort. Breast Cancer Res.

[85] Provenzano PP, Inman DR, Eliceiri KW, Knittel JG, Yan L, Rueden CT (2008). Collagen density promotes mammary tumor initiation and progression. BMC Med.

[86] Esbona K, Inman D, Saha S, Jeffert J, Schedin P, Wilke L (2016). COX-2 modulates mammary tumor progression in response to collagen density. Breast Cancer Res.

[87] Ostuni R, Kratochvill F, Murray PJ, Natoli G (2015). Macrophages and cancer: from mechanisms to therapeutic implications. Trends Immunol.

[88] Franklin RA, Liao W, Sarkar A, Kim MV, Bivona MR, Liu K (2014). The cellular and molecular origin of tumor-associated macrophages. Science.

[89] Lin Y, Xu J, Lan H (2019). Tumor-associated macrophages in tumor metastasis: biological roles and clinical therapeutic applications. J Hematol Oncol.

[90] Ojalvo LS, King W, Cox D, Pollard JW (2009). High-density gene expression analysis of tumor-associated macrophages from mouse mammary tumors. Am J Pathol.

[91] Lin EY, Li JF, Bricard G, Wang W, Deng Y, Sellers R (2007). Vascular endothelial growth factor restores delayed tumor progression in tumors depleted of macrophages. Mol Oncol.

[92] Weichand B, Popp R, Dziumbla S, Mora J, Strack E, Elwakeel E (2017). S1PR1 on tumor-associated macrophages promotes lymphangiogenesis and metastasis via NLRP3/IL-1beta. J Exp Med.

[93] Welm AL, Sneddon JB, Taylor C, Nuyten DS, van de Vijver MJ, Hasegawa BH (2007). The macrophage-stimulating protein pathway promotes metastasis in a mouse model for breast cancer and predicts poor prognosis in humans. Proc Natl Acad Sci USA.

[94] Maggiora P, Marchio S, Stella MC, Giai M, Belfiore A, De Bortoli M (1998). Overexpression of the RON gene in human breast carcinoma. Oncogene.

[95] Eyob H, Ekiz HA, Derose YS, Waltz SE, Williams MA, Welm AL (2013). Inhibition of ron kinase blocks conversion of micrometastases to overt metastases by boosting antitumor immunity. Cancer Discov.

[96] Ekiz HA, Lai SA, Gundlapalli H, Haroun F, Williams MA, Welm AL (2018). Inhibition of RON kinase potentiates anti-CTLA-4 immunotherapy to shrink breast tumors and prevent metastatic outgrowth. Oncoimmunology.

[97] Wculek SK, Malanchi I (2015). Neutrophils support lung colonization of metastasis-initiating breast cancer cells. Nature.

[98] Ohs I, Ducimetiere L, Marinho J, Kulig P, Becher B, Tugues S (2017). Restoration of natural killer cell antimetastatic activity by IL12 and checkpoint blockade. Cancer Res.

[99] Stockmann C, Doedens A, Weidemann A, Zhang N, Takeda N, Greenberg JI (2008). Deletion of vascular endothelial growth factor in myeloid cells accelerates tumorigenesis. Nature.

[100] Allen E, Jabouille A, Rivera LB, Lodewijckx I, Missiaen R, Steri V, et al. Combined antiangiogenic and anti-PD-L1 therapy stimulates tumor immunity through HEV formation. Sci Transl Med. 2017;9: 10.1126/scitranslmed.aak9679 10.1126/scitranslmed.aak9679 PMC5554432 28404866

[101] Mazzieri R, Pucci F, Moi D, Zonari E, Ranghetti A, Berti A (2011). Targeting the ANG2/TIE2 axis inhibits tumor growth and metastasis by impairing angiogenesis and disabling rebounds of proangiogenic myeloid cells. Cancer Cell.

[102] Welford AF, Biziato D, Coffelt SB, Nucera S, Fisher M, Pucci F (2011). TIE2-expressing macrophages limit the therapeutic efficacy of the vascular-disrupting agent combretastatin A4 phosphate in mice. J Clin Invest.

[103] Kesavan R, Frömel T, Zukunft S, Laban H, Geyer A, Naeem Z (2020). Cyp2c44 regulates prostaglandin synthesis, lymphangiogenesis, and metastasis in a mouse model of breast cancer. Proc Natl Acad Sci USA.

[104] Wagner K-U. Models of breast cancer: quo vadis, animal modeling? Breast Cancer Res. 2003;6. 10.1186/bcr723 10.1186/bcr723 PMC314446 14680483

[105] Forrester E, Chytill A, Bierie B, Aakre M, Gorska AE, Sharid-Afshar A (2005). Effect of conditional knockout of the type II TGFB receptor gene in mammary epithelia on mammary gland development and polyomavirus middle T antigen induced tumor formation and metastasis. Cancer Res.

[106] Arun G, Diermeier S, Akerman M, Chang KC, Wilkinson JE, Hearn S (2015). Differentiation of mammary tumors and reduction in metastasis upon Malat1 lncRNA loss. Genes Dev.

[107] Vasiljeva O, Papazoglou A, Kruger A, Brodoefel H, Korovin M, Deussing J (2006). Tumor cell-derived and macrophage derived cathepsin B promotes progression and lung metastasis of mammary cancer. Cancer Res.

[108] Harris IS, Trekoar AE, Inoue S, Sasaki M, Gorrini C, Lee KC (2015). Glutathione and thioredoxin antioxidant pathways synergize to drive cancer initiation and progression. Cancer Cell.

[109] Li J, Karaplis AC, Huang DC, Siegel PM, Camirand A, Yang XF (2011). PTHrP drives breast tumor initiation, progression and metastasis in mice and is a potential therapy target. JCI.

[110] Pützer BM, Hitt M, Muller WJ, Emtage P, Gauldie J, Graham FL (1997). Interleukin 12 and B7-1 costimulatory molecule expressed by an adenovirus vector act synergistically to facilitate tumor regression. Proc Natl Acad Sci USA.

[111] Movahedi K, Schoonooghe S, Laoui D, Houbracken I, Waelput W, Breckpot K (2012). Nanobody-based targeting of the macrophage mannose receptor for effective in vivo imaging of tumor-assoiated macrophages. Cancer Res.

[112] Shiao SL, Ruffell B, DeNardo DG, Faddegon BA, Park CC, Coussens LM (2015). TH2-Polarized CD4(+) T Cells and Macrophages Limit Efficacy of Radiotherapy. Cancer Immunol Res.

[113] Nakasone ES, Askautrud HA, Kees T, Park JH, Plaks V, Ewald AJ (2012). Imaging tumor-stroma interactions during chemotherapy reveals contributions of the microenvironment to resistance. Cancer Cell.

